# The Role of Physiotherapists in Smoking Cessation Management: A Scoping Review

**DOI:** 10.3390/healthcare11030336

**Published:** 2023-01-23

**Authors:** Mohammad Z. Darabseh, Aseel Aburub, Eman E. Fayed

**Affiliations:** 1 Department of Physiotherapy, Faculty of Allied Medical Sciences, Al-Ahliyya Amman University, Amman 19328, Jordan; 2 School of Allied Health Professions, Keele University, Keele ST5 5BG, UK; 3 Austin Campus, University of St. Augustine for Health Sciences, Austin, TX 78739, USA; 4 Department of Physical Therapy for Cardio-Pulmonary Dysfunctions & Geriatrics, Misr University for Science and Technology, 6th of October 12573, Egypt

**Keywords:** physiotherapy, smoking cessation, counselling, patient education, rehabilitation programme, healthcare, tobacco prevention

## Abstract

Physiotherapy (PT) is a profession that includes education and close contact for long periods of time with patients for treatment sessions. Globally, smoking is prevalent and is expected to increase in the next decades; thus, smoking cessation (SC) is an important management strategy to mitigate further escalation. Little is known about PT practice in SC, and therefore, this study aimed to systematically review and discuss the published literature about the role of physiotherapists in smoking cessation management, opinions, and prevalence of SC counselling in physiotherapy practice; and to explore barriers towards smoking cessation counselling within physiotherapy practice. A systematic search was conducted through EBSCO, and articles were included if they assessed the role of PTs in SC management. The databases were searched for studies published between 1 January 1970 to 1 April 2022. Articles were excluded if they did not include PTs, if they did not include assessment of SC management/counselling, if they were not cross-sectional studies, if they were not written in the English language, or if they were conference abstracts. Seven studies were included in the review. The search identified no studies that have investigated the role of PTs in vaping cessation. It was found that PTs are not addressing SC counselling and management enough in their practice. In addition, the search revealed that lack of training, time, and knowledge are the most common barriers against including SC counselling in physiotherapy practice and rehabilitation programs. Exploring possibilities of including SC counselling according to the clinical guidelines is encouraged. Additionally, establishing solutions to overcome barriers against SC counselling as part of physiotherapy practice is essential.

## 1. Introduction

Despite the exceptional challenges brought on by the Coronavirus Disease 19 (COVID-19) pandemic in 2020, the cigarette smoking epidemic continues to be one of the greatest public health threats the globe has ever encountered, leading to death of over eight million people every year around the world [1]. On the other hand, 1.2 million are the result of indirect tobacco use [1]. In 2016, 77,900 deaths in the United Kingdom (UK) were caused directly or indirectly by smoking [2]. The United Kingdom’s Annual Population Survey estimates that, in 2019, 14.1% of people aged 18 years and above smoked cigarettes, which equals to 6.9 million people in the population [3]. However, the proportion of current smokers in the UK has fallen significantly from 14.7% in 2018 to 14.1% in 2019 [3]. In the United States (US), smoking remains the leading cause of preventable disease, disability, and death [4]. According to the Centres for Disease Control and Prevention, smoking causes more than 480,000 deaths each year, or one in five [5].

The great mass of literature on the effects of smoking on health has left no doubt that smoking is a major preventable cause of morbidity and mortality. Chronic obstructive pulmonary disease, emphysema, lung cancer, and cardiovascular disease continue to be the most common health problems associated with smoking cigarettes [5]. For example, smokers had lower respiratory functions and higher carboxyhaemoglobin than non-smokers, which may cause a degree of airway obstruction [6]. Smoking causes a reduction in exercise capacity that is not just due to a reduction in aerobic capacity but also due to an increase in the metabolic cost of breathing [7,8]. Smoking increases blood pressure and heart rate and increases the risk for atherosclerosis [8,9].

Smoking cessation (SC), usually called quitting smoking or stopping smoking, which is the process of discontinuing tobacco smoking. SC has a number of potential health benefits, including decreased future risks of tobacco-related diseases, slower progression of existing diseases caused by tobacco, and an increase in life expectancy by a minimum of 10 years [10]. The habit of smoking is related to nicotine dependence, which makes smoking cessation very difficult [9].

Cessation medications approved by the US Food and Drug Administration and behavioural counselling can greatly increase the likelihood of successfully quitting, especially when used together [11]. Combining nicotine replacement therapy with cessation medications increases the likelihood of successfully quitting [11]. Despite being effective and easy to implement, smoking cessation interventions are not widely used in primary and secondary care. Managing smoking cessation needs to become a key part of routine practice for all clinicians. It has been proven that standard smoking cessation treatments, with pharmacological and behavioural support, are effective [12], but the success rates remain low, with only less than 30% of smokers stopping smoking successfully [13,14].

Smoking cessation process includes five consecutive steps: precontemplation, contemplation, preparation, action; and maintenance [15]. In the Pre-contemplation step smokers are not seriously thinking about SC in the next six months. Subsequently, they need to be encouraged to think about the long-term and short-term negative effects of smoking. In the contemplation step, smokers are considering quitting within the next six months. In this step, clinicians need to focus on motivational interviewing, investigate relevant health effects of smoking use and explore SC barriers. Additionally, clinicians need to find out other physical and mental health factors that could affect adherence to the SC intervention. In the preparation step, smokers are considering to quit within the next month and have made at least one attempt to quit during the last year. In the action step, previous smokers who have quit during the last six months are at a high risk of relapses. In the last step, maintenance previous smokers who quit over the last six months need to use behavioral therapy to reduce the chances of relapse over time. 

The progression of SC depends on what stage they are in before the start of the intervention [15]. The steps could be easily by asking assessment questions. After allocating the step of the SC the patient in, clinicians could then math the best intervention according to the step. Previous literature reported that SC would be more successful by at least six months when matching the step with the intervention [15]. In all of the five steps, advising patients about the disadvantages of smoking is essential. In addition, encouraging and motivating patients in re-evaluation sessions could help in further progression of the cessation process [16]. Moreover, the stage-matched, patient-centered counseling programmes can help clinicians to be more confident and feel more effective in their prescribed SC intervention [16]. 

Smoking cessation guidelines also recommend using the 5A’s model: ask (screen for smoking), advise (provide a quit message), assess (evaluate readiness to quit), assist (provide treatment), and arrange (track cessation progress) [17]. Understanding the five steps and using the 5 A’s approach is essential for all clinicians in general, who are working in SC. From here it appears that clinician (for example: physiotherapists) who are involved in SC must be trained and have the required skills and knowledge in all of the processes of smoking cessation.

Physiotherapy (PT) outcomes can be seriously impaired in people who smoke, either through the direct effects of smoking on organ systems [14,18] or through the vasoconstrictive and hypoxic effects of nicotine, leading to impaired perfusion and delayed tissue healing and repair [19,20].

Although increasing level of physical activity, health education is endorsed as a smoking cessation aid [12,21], and most physiotherapists (PTs) adopted the view that advising people who smoke to quit is a clinical responsibility and recommended greater involvement of the profession in helping people who smoke quit [12], Canadian physiotherapists are not routinely involved in smoke cessation plans. Moreover, the new health promotion models mandate physiotherapists to be well equipped with knowledge and skills to encourage smoking cessation among physiotherapists, patients, and wider community [22]. Because physiotherapists spend a long time treating patients for many sessions, it could be for years in some cases, and the trusting relationships they have with patients; their educational background; and their wide range of practice across, including SC as one of physiotherapist roles, could be of importance [23]. In fact, there are only 50% of Canadian physiotherapists who are informed about the clinical guidelines for smoke cessation counselling, and only 30% of physiotherapists received smoke cessation counselling training [22]. 

Perhaps, lack of training and time prevents physiotherapists from practicing their role to keep people healthy and active, with or without the presence of injury or disease [22]. Physiotherapists should use their practice pattern of seeing patients’/clients’ multiple visits over a prolonged period of time not only to address their impairments but also by education pillar through providing smoke cessation counselling when needed [24]. 

This study aimed to systematically review and discuss the published literature about the role of physiotherapists in smoking cessation management, opinions, and prevalence of SC counselling in physiotherapy practice; and to explore barriers towards smoking cessation counselling within physiotherapy practice. 

## 2. Method

### 2.1. Design

The study was designed to provide a scoping review and narrative synthesis of relevant published literature. A Preferred Reporting Items for Systematic reviews and Meta-Analyses extension for Scoping Reviews (PRISMA-ScR) [25] was used to ensure reporting of the required information. 

### 2.2. Study Protocol

The protocol of this review is registered in the International prospective register of systematic reviews database (PROSPERO) (registration number: CRD42021278593).

### 2.3. Search Strategy

A search was conducted through Elton B. Stephens Company (EBSCO) using the following electronic databases: Medical Literature Analysis and Retrieval System Online (MEDLINE), Allied and Complementary Medicine Database (AMED), SPORTDiscus, and Cumulative Index to Nursing and Allied Health Literature (CINAHL). These databases were selected because they might contain published articles about physiotherapy. Additionally, another search was performed using the PEDro database (the Physiotherapy Evidence Database). The search was limited to full-text English-language articles and excluded conference abstracts. The selected databases were chosen because of the likely availability of physiotherapy- and exercise-related articles in these databases. Backward snowballing was conducted by hand-searching the reference lists of included studies to identify other potentially relevant studies.

The databases were searched for studies published between 1 January 1970 to 1 April 2022, with the results of the searches managed using Endnote Version X7 (Clarivate Analytics, Philadelphia, PA, USA).

#### 2.3.1. Keywords Used

Both medical subject heading (MeSH) terms and free-text terms were used to construct the search algorithm. Keywords used were structured by using the PICO approach (population, intervention, comparison, and outcome) [26]. Table 1 summarizes the combinations of keywords included in the search strategies. PICO search terms were combined using Boolean operators ‘AND’ and ‘OR’ (Table 1). Keywords used were: “Physiotherapy” OR “physiotherapist” OR “physiotherapists” OR “physical therapy” OR “Physical therapists” OR “Physical therapist”; AND “smoking cessation” OR “smoking cessation interventions” OR “quit smoking” OR “stop smoking” OR “tobacco cessation” OR “smoking abstinence” OR “vape” OR “vaping” OR “e-cigarette” OR “e-cig” OR “electronic cigarette”.

#### 2.3.2. Inclusion/Exclusion Criteria for the Articles

Articles were included if they assessed the role of physiotherapists in SC management. Articles were excluded if they did not include PTs, if they did not include assessment of SC management/counselling, if they were not cross-sectional studies, if they were not written in the English language, or if they were conference abstracts.

#### 2.3.3. Study Selection

Following the search and subsequent removal of duplicates, titles or abstracts were screened for relevance. Full texts of relevant studies were then screened for eligibility against inclusion and exclusion criteria.

#### 2.3.4. Data Extraction

The following data were extracted from the included studies and presented in Table 2: author (year); sample size; study design, study goals; and key findings. 

## 3. Results

**Figure 1:**
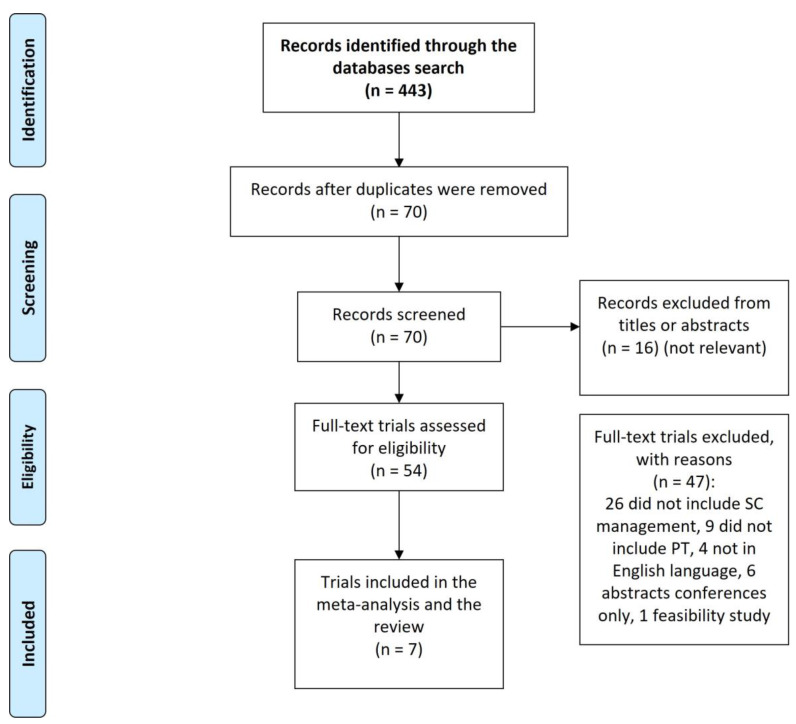
PRISMA flowchart for the search records and the included studies.

The systematic search identified 443 citations from EBSCO databases, of which 373 citations were duplicates. Consequently, 70 citations were screened from titles or abstracts, and 16 were considered not to be relevant. Of the 54 remaining studies, 47 articles were excluded: 26 were excluded because they did not include SC management/counselling, 9 did not include PT, 4 studies were excluded because they were not written in the English language, 6 were abstract conferences only, and one study was a feasibility study to pilot a new curriculum that includes SC in PT entry-level academic programmes (Table 2). Consequently, seven studies were included in the review. Figure 1 below represents the PRISMA flowchart for the included/excluded search records. The search identified no studies that have investigated the role of PTs in vaping cessation (Table 2). Table 2 is the data extraction table for the included seven studies. Three of the included studies (Bonder et al. 2011; Bonder et al. 2014; and Bonder et al. 2020) are from one big research project and have the same authors, similar titles, and sample, but with different goals in each. 

### 3.1. Opinions of PTs about Including SC Counselling in Their Practice

Bodner et al. (2011) reported that 76% of the PTs strongly agreed that physical therapists should ask their patients about smoking habits, and 51.9% of PTs agreed that PTs should be more involved in SC counselling (Table 2). Similarly, another study reported that 73% of PTs agree that all patients should be screened for tobacco use and that PTs should provide SC counselling for patients who smoke [31] (Table 2). Moreover, most of PT programme directors (84%) at universities agreed that physiotherapists should screen patients for tobacco use, and 81% of them reported that students should be trained in screening patients for tobacco use [30] (Table 2).

### 3.2. Prevalence of SC Counselling in Physiotherapy Practice

The prevalence of including SC counselling in PT practice was assessed in four studies [22,28,29,31]. Bonder et al. (2011) reported that only 65% of PTs were actually addressing SC in their practice. In another study, around 54% of PTs counsel rarely or not at all, and only 21.6% of PTs reported assisting their patients to quit smoking [28] (Table 2). Surprisingly, Pignataro (2017) reported that only 13% of PTs assess nicotine addiction, 31% of PTs recommend SC on a consistent basis, and 48% do so on an occasional basis. McCleary et al. (2012) identified that only PTs who completed SC counselling training (30%) include SC management in their practice and continue SC follow-up with their patients (Table 2). 

### 3.3. Barriers to Including SC Counselling in PTs Practice

#### 3.3.1. Lack of Knowledge 

Balfour (1993) investigated the level of knowledge of PTs about pathological, physiological, and anatomical changes in the respiratory system; and respiratory diseases that smoking is a strong predisposing factor. Balfour reported that answers of PTs who were surveyed demonstrate a poor level of knowledge about the harmful effects of smoking on the respiratory system, and the level of knowledge was not associated with the number of years worked with patients with medical respiratory problems [27]. On the other hand, Bonder et al. (2011) reported that most PTs who were surveyed have good knowledge about the effects of smoking on the prevalence of respiratory and cardiac diseases (Table 2). 

When asked about the knowledge of PTs about SC counselling guidelines, different findings were reported in the included studies. McCleary et al. (2012) reported that around 50% of PTs know about the clinical guidelines for SC counselling. Whereas Bonder et al. (2012) reported that only 1.6% of PTs applied the 5A’s approach for SC, and Pignataro et al. (2017) reported around 6% of PTs were familiar with the 5A’s approach for SC counselling. When assessing coverage of SC guidelines in PT programmes, Pignataro et al. (2014) found that only 40% of programmes include skills based on clinical practice guidelines regarding SC counselling (Table 2).

#### 3.3.2. Lack of Training 

All seven included studies revealed a lack of training of PTs for SC counselling. For example, Bonder et al. (2011) reported that only 1.7% of PTs are well prepared and trained to be involved in SC counselling, and the majority (71.6%) of the PTs reported not being at all prepared to provide counselling. Similarly, McCleary et al. (2012) reported that only 30% of PTs received SC counselling training. Both Pignataro et al. (2017) and Balfour (1993) reported a lack of training as the main barrier to not including SC counselling in PT practice. When PT academic programmes were assessed for inclusivity of SC counselling skills within clinical sittings training, only 10% of the programmes included SC counselling skills practice within a clinical setting [30] (Table 2).

#### 3.3.3. Lack of Time

The time factor was reported in two studies as a barrier to including SC counselling in PT practice [22,31] (Table 2). 

#### 3.3.4. Other Barriers 

Other barriers that were addressed by the results of the included studies include the perception of PTs that SC is a professional role and the feeling of PTs about self-efficacy to counsel [32] (Table 2). 

The key characteristics of patients that PTs perceived as barriers to SC counselling were patients’ lack of adherence, lack of long-term commitment, and emotional or psychological status [22] (Table 2). 

## 4. Discussion

To our knowledge, this is the first scoping review that addresses the role of PTs in SC management. This scoping review revealed that PTs are not addressing SC counselling and management enough in their practice [22,28,29,31], although most PTs agreed about the need to address SC management and nicotine screening in their practice [22,30,31]. This scoping review identified no studies that have investigated the role of PTs in vaping cessation. It was found that PTs are not addressing SC counselling and management enough in their practice. In addition, the search revealed that lack of training, time, and knowledge are the most common barriers against including SC counselling in physiotherapy practice and rehabilitation programs.

The results of this review are similar to the findings of other studies about the role of allied health professions in general in smoking cessation. For example, Charesworth et al. (2021) reported the successful role of radiographers in smoking cessation. Additionally, the role of allied health professions was found to be successful, as reported in a previous study [33]. However, lack of time was reported as a barrier to smoking cessation counselling in those studies [33,34]. Furthermore, a lack of knowledge and training was reported in radiographers s barriers to smoking cessation [34]. Other barriers that have been mentioned by other healthcare providers include organization and infrastructure, patient requirements, and culture [34].

Smoking cessation clinical guidelines have been established for allied health professions since the 1990s [35,36,37,38]. The most common and widely recommended clinical guideline for SC include the 5As approach: (1) Ask about smoking status; (2) Advise briefly to quit; (3) Assess tobacco dependence and motivation to quit; (4) Assist with support and medication; (5) Arrange follow-up [35,36,37,38]. However, physiotherapists, similar to other allied health professions, might face some difficulties in applying this approach in their clinical practice. In our study, the systematic approach revealed that lack of training, time, and knowledge are the most common barriers against including SC counselling in physiotherapy practice [22,27,28,29,30,31,39]. Accordingly, universities and educational physiotherapy colleges around the world are strongly recommended to include SC guidelines in their curriculums and to include SC counselling skills in their practical and clinical skills that are essential for students. Additionally, we recommend hospitals and private physiotherapy centres provide training for PTs about SC management and its clinical guidelines. 

By increasing the number of physiotherapists trained in tobacco cessation counselling, more people with disabilities might be able to quit smoking, which might improve the outcomes of physiotherapy intervention. Physiotherapists are already highly skilled in patient education and outcome evaluation. It is likely that tailored tobacco cessation counselling training for Physiotherapists could achieve similar smoking cessation rates, particularly among people with disabilities. Moreover, follow-up arrangements that are performed by health professionals, including physiotherapists, can be implemented, given multiple interactions with patients and frequently weekly visits, often over weeks/months [22,30].

The number of people who smoke tobacco is expected to be over 1.1 billion worldwide in 2025 [40]. This huge prevalence of smokers will increase the health-related demands and costs of treatment for diseases caused by smoking, such as respiratory and cardiovascular diseases [40]. Accordingly, using every chance to decrease the number of smokers and encourage smoking cessation is essential. Hence, physiotherapy is a profession that includes education, close contact with patients/clients, and long periods and sessions of treatment, and physiotherapists are considered key players in health promotion and patient education [23]. 

## 5. Limitation

Limitations of this review include that it has only included seven studies and the potential for missing unpublished data. Additionally, the study also did not include quality assessment for the included studies.

## 6. Conclusions

This scoping review addressed the role of PTs in smoking cessation management. The review revealed that PTs are not addressing SC counselling and management enough in their practice, although most PTs agreed about the need to address SC management and nicotine screening in their practice. Exploring possibilities of including SC counselling according to the clinical guidelines is encouraged. Additionally, establishing solutions to overcome barriers and improving SC counselling as part of physiotherapy practice is essential. Furthermore, providing PTs with the needed trainings and courses to enhance their SC counselling for their patients/clients is highly recommended.

## Authors’ Contribution

All authors contributed to the study conception, design, and preparation. M.Z.D. and A.A. extracted the data. M.Z.D. wrote the first draft of the manuscript. All authors commented on previous versions of the manuscript. All authors read and approved the final manuscript.

## Figures and Tables

**Table 1:** Keywords and search strategy used, using the PICO approach in the selected databases.

Population	Intervention	Comparison	Outcome Measures
**“Physiotherapy” OR “physiotherapist” OR “physiotherapists” OR “physical therapy” OR “Physical therapists” OR “Physical therapist”**	**“Smoking cessation” OR “smoking cessation interventions” OR “quit smoking” OR “stop smoking” OR “tobacco cessation” OR “smoking abstinence” OR “vape” OR “vaping” OR “e-cigarette” OR “e-cig” OR “electronic cigarette”.**	**No physiotherapy intervention**	**Success of cessation**

**Table 2:** Data extraction table for the included articles (*n* = 7).

Author (Year)	Sample Size	Study Design	Study Goals	Key Findings
Balfour (1993) [27]	63 PTs	Cross-sectional postal survey	Describe the level of knowledge of PTs about the physiological effects of smoking. Assess if amount of knowledge is related to duration of experience in general and in respiratory care or to grade and seniority. Determine if there is a need to educate PTs about the effects of smoking.	Poor level of knowledge about the effects of smoking among PTs. The number of years worked as a PT and the number of years of working with patients with medical respiratory problems did not affect the levels of knowledge. PTs agreed that they need more education and training about smoking and SC management.
Bonder et al. (2011) [22]	738 PTs	Cross-sectional postal survey	To assess PTs’ knowledge about the health effects of smoking. To assess PTs’ views about addressing SC counselling in practice. To assess barriers to addressing SC counselling in physiotherapy management.	98.2% of PTs strongly agreed that passive smoking increases the risk of lung diseases. 80.1% of PTs agreed that passive smoking increases the risk of heart diseases. 76% of the PTs strongly agreed that physiotherapists should ask their patients about smoking habits. Only 65% of PTs were actually addressing SC in their management. 51.9% of PTs agreed that PTs should be more involved in SC management. Only 1.7% of PTs reported that they are well prepared to be involved in SC counselling. The majority (71.6%) of the PTs reported not being at all prepared to provide counselling. The main barriers to helping patients in SC included lack of resources for providing SC counselling and lack of time. The key characteristics of patients that PTs perceived as barriers to SC counselling were patients’ lack of adherence, lack of long-term commitment, and emotional or psychological status.
Bonder et al. (2012) [28]	738 PTs	Cross-sectional postal survey	To examine SC counselling practices of PTs, including the frequency of such counselling. To assess if PTs use the established 5A’s approach (ask, advise, assist, assess, and arrange follow-up). To assess if SC training was received.	Most PTs (54.0%) counsel rarely or not at all. Only 21.6% of PTs reported assisting their patients to quit smoking. Only 1.6% of PTs reported that they applied the 5A’s. Only 12.4% of PTs reported receiving SC counselling training.
McCleary et al. (2012) [29]	602 PTs	Cross-sectional postal survey	To assess PTs knowledge and uptake about the Ask, Brief, and Cessation (ABC) approach for SC counselling.	Around 50% of PTs know about the clinical guidelines for SC counselling. Only 30% of PTs received the SC counselling training. PTs who completed the training were including SC counselling and follow-up with their patients.
Pignataro et al. (2014) [30]	146 PT programme directors	Cross-sectional online survey	To assess the need for SC training within entry-level PTs education. To identify potential barriers to implementation of SC guidelines in the PTs curriculums.	60% of entry-level PT education programmes included student training on how to provide SC counselling. 40% of programmes included skills based on clinical practice guidelines regarding SC counselling. Training for screening patients for tobacco use and nicotine addiction was present in 67% and 40% of the programmes, respectively. Few programmes (10%) included SC counselling skills practice within a clinical setting. Most of programme directors (84%) agreed that physiotherapists should screen patients for tobacco use, and 81% of them reported that students should be trained in screening patients for tobacco use.
Pignataro et al. (2017) [31]	212 PTs	Cross-sectional online survey	To assess the prevalence of PT SC counselling and barriers toward implementation of SC in PTs management programmes.	Around 6% of PTs were familiar with the 5A’s approach. 80% of PTs lack training in SC guidelines, counselling, and management. Only 13% of PTs assess nicotine addiction. 31% of PTs recommend cessation on a consistent basis, and 48% do so on occasional basis. 28% of PTs reported that there might not be enough time to provide SC counselling. 73% of PTs reported that all patients should be screened for tobacco use and that PTs should provide cessation counselling for patients who smoke. Lack of knowledge and training for SC counselling were the main barriers to including SC counselling in PTs management.
Bonder et al. (2020) [32]	738 PTs	Cross-sectional postal survey	To assess factors associated with PTs’ intentions to counsel for SC, including self-efficacy, moral agency, and environmental factors.	PTs who have the perception that SC counselling is a professional role had greater intention to counsel. Increased self-efficacy to counsel for SC is associated with Having the skills and knowledge to counsel.

## Data Availability

Data is contained within the article.
